# Is one-stage lateral sinus lift and implantation safe in severely atrophic maxillae? Results of a comparative pilot study

**DOI:** 10.1186/s40729-023-00471-5

**Published:** 2023-02-19

**Authors:** Sascha Virnik, Laura Cueni, Anita Kloss-Brandstätter

**Affiliations:** 1 Department of Oral and Maxillofacial Surgery, Clinic Klagenfurt Am Wörthersee, 9020 Klagenfurt, Austria; 2 Department of Engineering and IT, Carinthia University of Applied Sciences, Europastraße 4, 9524 Villach, Austria

**Keywords:** Maxillary sinus floor augmentation, Lateral window approach, One-stage sinus lift, Residual bone height < 3 mm, Implant survival

## Abstract

**Purpose:**

The aim of this retrospective comparative study was to evaluate the survival of dental implants placed in the posterior maxilla with a residual bone height less than 3 mm using a one-stage lateral sinus lifting approach. The research question was whether in very severely atrophied maxillary bones (residual height < 3 mm), a sinus lift with simultaneous implant placement would be associated with a higher complication rate compared to single-stage sinus lifts at average residual alveolar process heights.

**Methods:**

Complications of 63 implants, where the residual bone height was below 3 mm, were compared to a reference group of 40 implants, which were inserted using a one-stage lateral sinus lift in maxillae with at least 3 mm residual bone height. Implant survival, bleeding-on-probing, the presence of peri-implant mucositis and the occurrence of peri-implantitis were documented.

**Results:**

The mean follow-up time for implant survival was 80.3 ± 25.9 months. One implant out of 63 was lost in the severely atrophic maxilla group and two implants out of 40 were lost in the reference group. There were no differences in the occurrence of implant loss (*p* = 0.558), bleeding-on-probing (*p* = 0.087), peri-implantitis (*p* = 0.999) and peri-implant mucositis (*p* = 0.797) between the severely atrophic alveolar ridge group and the reference group.

**Conclusions:**

Even in severely atrophic maxillae with < 3 mm residual bone height, a one-stage maxillary sinus lift and immediate implant placement can be carried out safely.

**Graphical Abstract:**

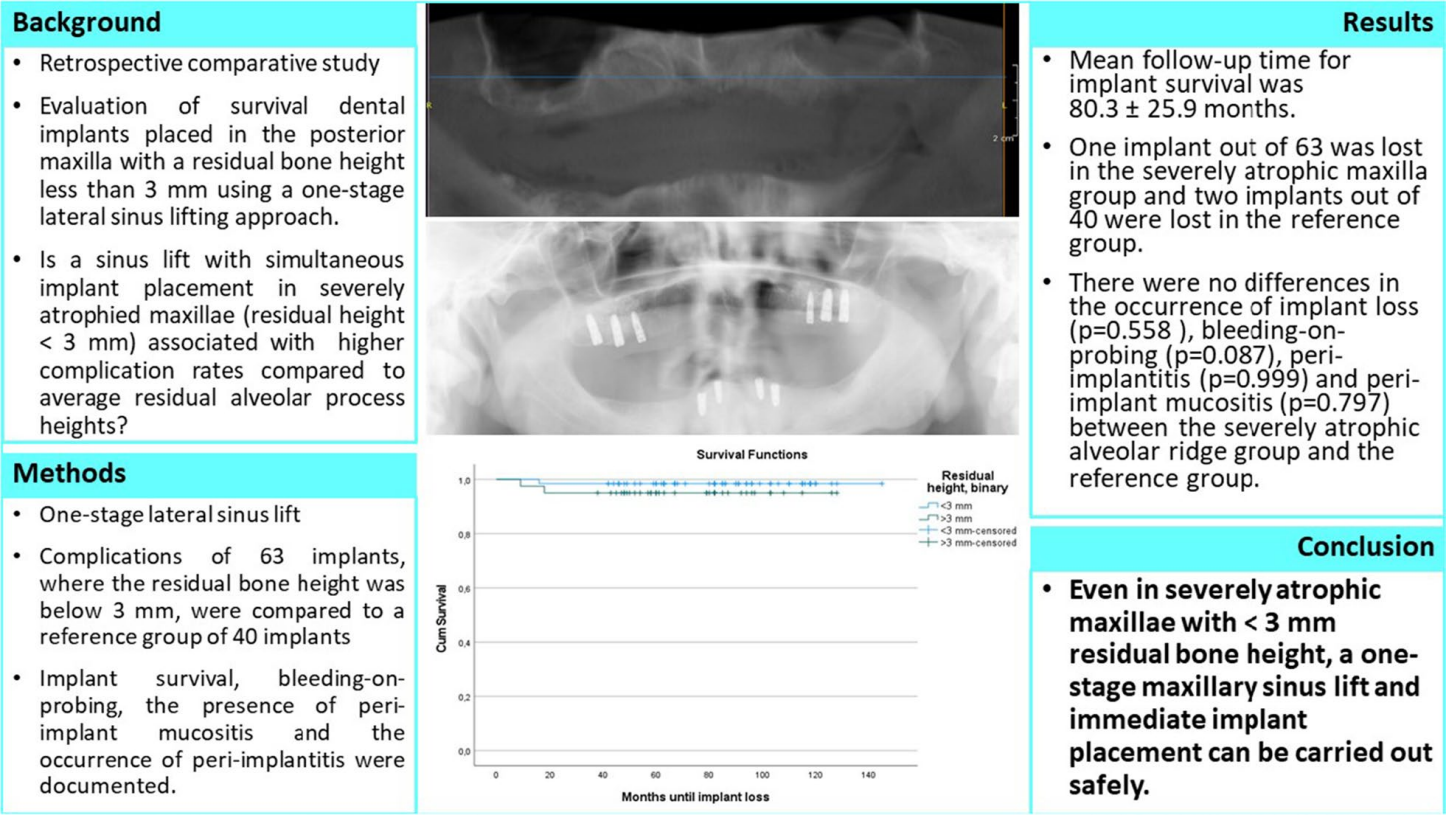

## Background

Implant therapy in the atrophic posterior upper jaw becomes a challenge with reduced maxillary bone height. If the height of the remaining alveolar bone is less than 6 mm, sinus augmentation is recommended to correct this condition before inserting the implant [1].

For maxillary sinus augmentation and implant placement, there are three surgical options: two-stage lateral sinus augmentation, single-stage lateral sinus augmentation (with simultaneous implant placement), and one-step crestal approach with simultaneous implant placement, each with pros and cons [2].

The procedure of sinus augmentation (also called sinus lift, sinus graft, or sinus procedure) is a surgical procedure in which the Schneiderian membrane is lifted from the surrounding sinus wall and a bone graft is placed underneath it. By applying the sinus lift using the lateral window technique with a grafting material, the severely atrophic posterior maxilla can be supplied with dental implants safely and predictably with a low incidence of morbidity [3]. Indeed, Schiegnitz and colleagues evaluated the oral health-related quality of life after sinus augmentation and found a remarkable benefit for the patients through this procedure [4].

Sinus augmentations are intended to provide bone to support dental implants. Synchronous placement can be performed at the same time as sinus surgery ("one stage procedure") or delayed placement can be performed after a period of healing [3]. Until now, the decision to use one- or two-stage procedures was made primarily on the amount of remaining bone and the potential of providing primary stability for the placed implants. If the height of the residual alveolar bone allows for sufficient primary implant stability, dental implants are placed at the same time as the augmentation treatment. If not, depending on the graft material utilized, the implants are implanted 4 to 12 months after the augmentation surgery [3].

Current recommendations for maxillary sinus augmentation considering the degree of atrophy state that for a residual bone height of 4–6 mm, a one-stage sinus lift with lateral access and bone graft and immediate placement of implants can be safely applied. However, for a residual bone height of 1–3 mm, the guidelines recommend a maxillary sinus elevation with lateral access and application of bone grafts, but with a deferred placement of implants [5, 6].

The aim of this study was to determine the implant survival rate and potential complications of maxillary sinus augmentation applying the lateral window technique with a grafting material and immediate implant placement in patients with a crestal bone height of < 3 mm. The reason for falling below the recommended minimum bone height for simultaneous implantation is to spare the patient a second surgical procedure with postoperative pain. In order to set the results into the context of standard one-stage sinus lift and implantation, the data obtained from severely atrophic maxillae were compared to data from maxillae with a residual height of at least 3 mm.

The primary outcome of this study was the survival of the implants. The secondary outcomes were the occurrence of bleeding-on-probing, the probing depth (mm), the onset of peri-implantitis, the appearance of mucositis and the incidence of membrane perforation. The null hypothesis was that there were no differences in the primary and secondary outcome variables between severely atrophic maxillae and maxillae with a residual height of at least 3 mm.

## Materials and methods

### Patients

This monocentric study comprises the cases of 63 patients (40 females and 23 males), who were candidates for maxillary sinus floor augmentation and simultaneous implant placement. The patients were fully informed about the surgical procedures, treatment alternatives, aim and design of the study and signed a written consent form before surgery. The local ethical committee agreed on the conduction of the study (EK-Nr.: S2020-21). The patients were operated between September 2009 and February 2018. All patients were followed up clinically in October 2021.

Inclusion criteria were as follows: posterior edentulous subjects (regions 15–17 and 25–27) without active periodontal diseases. In all cases, the alveolar bone ridge was wide enough for simultaneous implant placement.

Exclusion criteria were as follows: high tobacco consumption (> 20 cigarettes/day), untreated diabetes, untreated periodontitis, systemic disease that would contraindicate oral surgery, and bad oral hygiene.

### Radiographic analysis

Panoramic radiographs were taken to map out the patient's upper jaw and sinuses. Every patient was subjected to three-dimensional X-ray diagnostics (digital volume tomography, or DVT in short), followed by computer-aided planning of the augmentation and immediate implantation (KaVo OP 300 Maxio DVT). The sinus' height was measured before the surgical procedure and at the final follow-up.

### Surgical procedure

The partially edentulous patients underwent a thorough initial periodontal examination, including the assessment of plaque, gingivitis, and probing depth. Immediately before the operation, the patients were instructed to rinse their mouth with 0.2% chlorhexidine mouthwash for 1 min.

The used surgical procedure was lateral window technique with simultaneous implant(s) insertion. The surgical intervention was performed under infiltration of local anesthetics (Ultracain DS forte, Sanofi-Aventis, Vienna, Austria). The height of the alveolar bone was measured intraoperatively with an Astra Tech probe from the implantology cassette (Dentsply Sirona Austria GmbH, Vienna, Austria).

The Caldwell–Luc procedure was used to access the maxillary sinus through the lateral wall. A mucosal midcrestal incision was performed with anterior and posterior releasing vestibular incisions certain distance from the proposed osteotomy site. A full-thickness flap was reflected to expose the lateral maxillary wall. An oval or round bony window was created with a diamond-coated round-bur so that the Schneiderian membrane became visible. The sinus membrane was elevated carefully with a sinus curette.

In total, 103 implants were inserted in the maxillae of 63 patients. Depending on the area of operation, the diameter of the implants varied between 3.0 and 4.2 mm, and the length of the implants varied between 9 and 11 mm.

Resorbable collagen membranes were used for coverage of the augmentation sites. The OsseoGuard membrane (Zimmer Biomet Austria GmbH, Vienna, Austria) was used for 85 implants, and the OSSIX® PLUS membrane (REGEDENT GmbH, Dettelbach, Germany) was used for 18 implants. The monofilament, non-absorbable Ethilon 5.0 suture material (Johnson & Johnson, New Brunswick, New Jersey, USA) was used for wound closure.

Routine postoperative care included administration of amoxicillin and clavulanic acid (1000 mg augmentin, administered orally, three times a day for 4 days), ibuprofen (600 mg, administered orally, every 6 h as needed), and mouthwash (0.2% chlorhexidine, three times daily for 7 days) [7].

### Implants and augmentation material

The inserted implants were from Astra (Type Astra Tech Implant System EV: *n* = 59; Type Astra Tech Implant System OsseoSpeed TX: *n* = 39; Dentsply Sirona Austria GmbH, Vienna, Austria) and from Straumann (Type Bone Level: *n* = 5; Straumann Holding AG, Basel, Switzerland). There were no differences in the implants inserted and the augmentation material used between the two study groups (Table 1).

**Table 1:** Diagnostic and surgical characteristics of the study population

	Residual height, binary		*p*-value
	< 3 mm [*n* = 63]	≥ 3 mm [*n* = 40]	
Height of residual bone (mm)			
Mean	2.198	3.89	< 0.001
Std. deviation	0.577	0.966	
Boneloss (mm)			
Mean	0.310	0.288	0.906
Std. deviation	0.931	0.9138	
Follow-up years			
< 1 year [*n* = 10]	6	4	0.232
1–2 years [*n* = 16]	6	10	
2–3 years [*n* = 18]	13	5	
3–4 years [*n* = 14]	6	8	
4–5 years [*n* = 15]	11	4	
5–6 years [*n* = 10]	6	4	
6–7 years [*n* = 13]	10	3	
7–8 years [*n* = 7]	5	2	
Type of augmentation material			
Xenogeneic [*n* = 70]	46	24	0.197
Autogenous [*n* = 33]	17	16	
Augmentation material			
Endobon [*n* = 48]	29	19	0.505
BioOss [*n* = 8]	6	2	
Osteopure [*n* = 6]	5	1	
Endobon + autogenous bone [*n* = 41]	23	18	
Type of membrane			
Osseoguard [*n* = 85]	55	30	0.120
Ossix [*n* = 18]	8	10	
Type of implant			
Astra EV [*n* = 59]	33	26	0.176
Astra TX [*n* = 39]	28	11	
Straumann [*n* = 5]	2	3	
Type of provisorium			
Crown [*n* = 66]	39	27	0.783
Bridge [*n* = 30]	19	11	
Complete denture [*n* = 7]	5	2	

*P*-values were obtained from *t*-tests when comparing metric variables or from Chi-squared tests when testing the independence of categorical variables

In all patients and implants, it was necessary to use xenogeneic bone substitution material for sinus floor augmentation. In 62 implants, the sinus lift was carried out with purely xenogeneic bone substitution material, while in 41 implants the augmentation was carried out with a mixture of xenogeneic material and autogenic bone. In 48 implants, the “window” of the sinus was filled with Endobon (Zimmer Biomet Austria GmbH, Vienna, Austria). A mixture of Endobon and autologous bone was inserted in 41 implants. BioOss (Geistlich Pharma AG, Wolhusen, Switzerland) was used for simultaneous augmentation in 8 implants, and OSTEOpure (European cell and tissue bank ECTB, Wels, Austria) was used for simultaneous augmentation in 6 implants.

### Prosthetic restoration

All implants were given at least 6 months for full osseointegration. There were four different prosthetic restorations: 66 implants received a single tooth crown, 30 implants had blocked crowns or implant bridges as a restoration, and seven implants were involved in complete prosthesis. Four implants were supplied with locators for anchoring the prosthesis, two implants had telescopes and two implants connected a bar construction for the prosthesis. There was no difference in the prosthetic restoration between the two study groups (Table 1; *p* = 0.783).

### Clinical follow-up investigation

On the day of the clinical follow-up, all 63 patients had X-rays taken in the form of a single tooth image and an orthopantomogram.

The patients were checked for the presence of mucositis [8] and peri-implantitis [9]. If peri-implantitis was present, the bone loss was visualized on the X-ray and measured using the X-ray program. The following criteria were used for the diagnosis of peri-implantitis: presence of bleeding and/or suppuration on gentle probing; increased probing depth compared with baseline examination; and presence of radiographic marginal bone loss ≥ 0.5 mm when compared with baseline radiograph [10, 11]. An implant loss was also noted.

The probing depth was measured at 6 points of each superstructure (mesiobuccal, buccal, distobuccal, distopalatinal, palatinal and mesiopalatinal).

### Statistical analyses

For this retrospective analysis, the dataset was divided into two study groups based on the residual height of the alveolar ridge: the severely atrophic groups with a residual height of less than 3 mm, and the reference group with a residual height of at least 3 mm (Table 1).

Statistical analyses were performed with IBM SPSS (version 27; International Business Machines Corp., Armonk, NY, USA). The data set was complete, and there were no missing data. For descriptive statistics, means and standard deviations were calculated. Pearson's Chi-squared test was applied to sets of unpaired categorical data to evaluate the probability that an observed difference between the datasets was due to chance. Fisher's exact test was used where sample sizes were small. An independent sample *t*-test was used when two separate sets of independent and identically distributed samples were obtained, and their population means were compared to each other.

A Kaplan–Meier analysis was used to estimate the probability that no losses occurred within a certain period of time for the implants examined [12]. A Mantel–Cox test was then used to check whether the two Kaplan–Meier survival curves (that of implants in extremely atrophied maxillae versus that of implants in average atrophied maxillae) were statistically different from each other [13].

Only two-sided significance tests were used. A probability of error of *p* ≤ 0.05 was chosen as the threshold value [14–16]. An alpha adjustment for multiple testing was not performed. The results are therefore explorative and descriptive.

## Results

### Documentation of one patient as an example for the surgical procedure

**Fig. 1:**
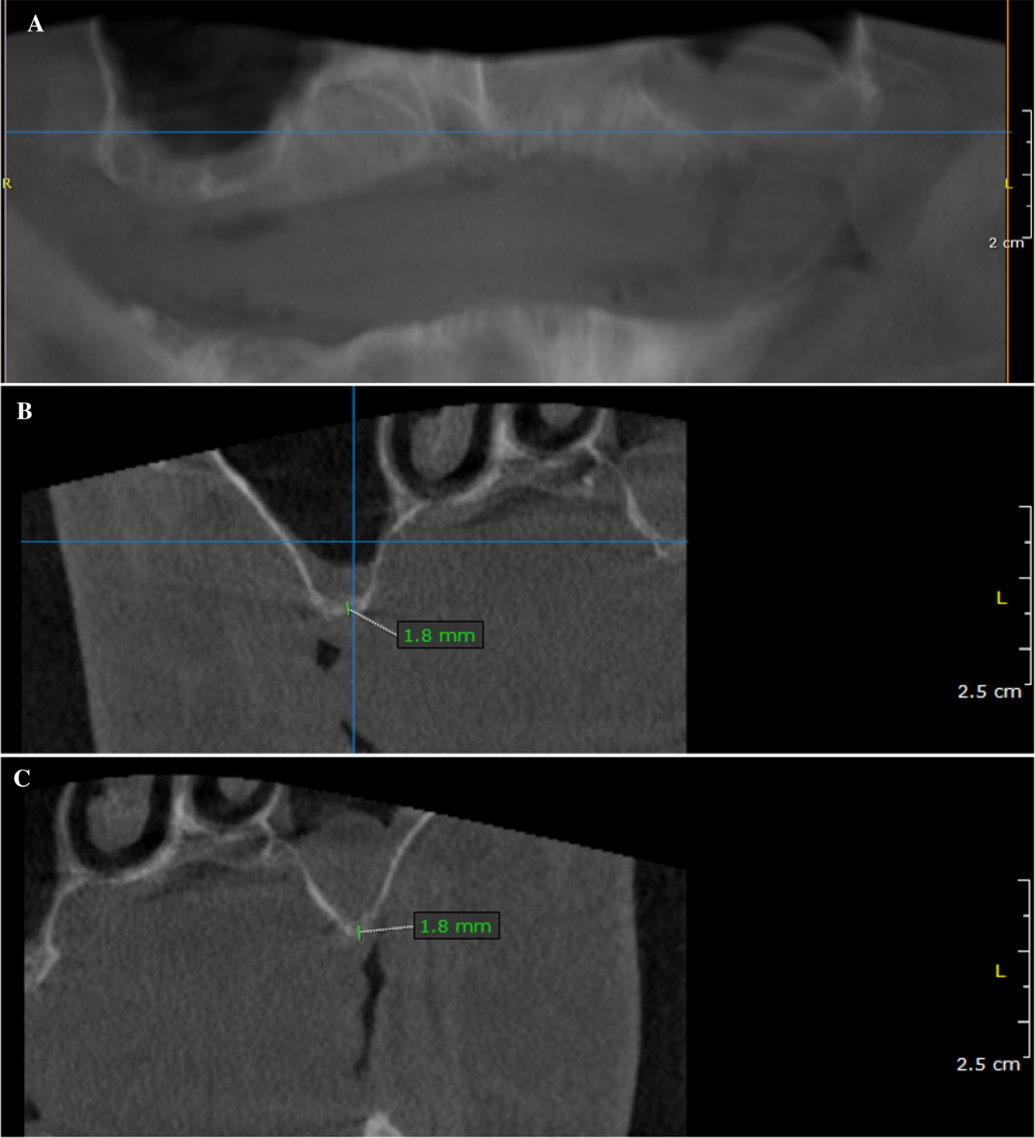
Radiographic evaluation of an alveolar ridge defect with a Type-III bone defect in the maxilla before augmentation. **A** Panoramic radiograph demonstrated massive bone loss in the right and left maxilla. The patient showed local swelling of the basal maxillary sinus mucosa during the preliminary examination. After assessment by an otorhinolaryngologist, the patient had a clinically asymptomatic situation and there was no objection to augmentation of the sinus. **B** Sagittal section of the CBCT illustrating the region of interest in the first quadrant. **C** Sagittal section of the CBCT illustrating the region of interest in the second quadrant. The green line corresponds to the vertical height of the bone in the defect area before augmentation

**Fig. 2:**
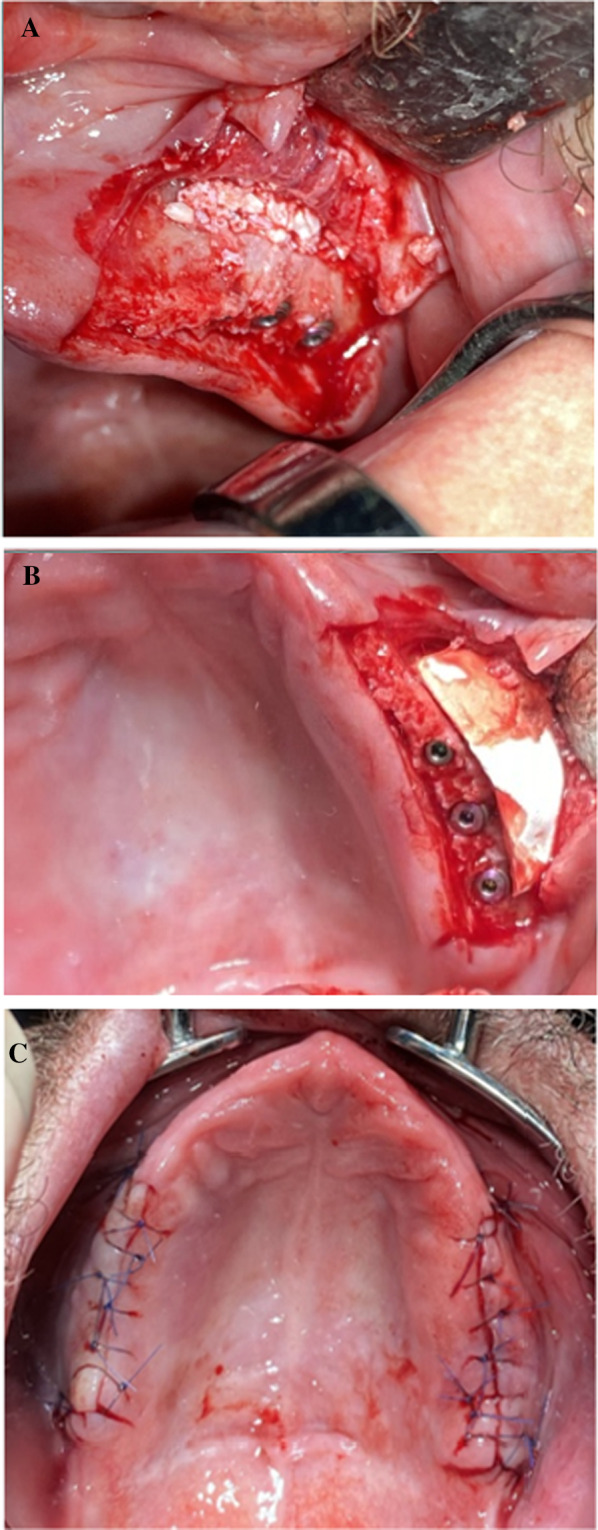
Intraoperative clinical situation during augmentation with simultaneous implantation. **A** Intraoperative situation after augmentation with additional application of bovine bone particles and implantation with the lateral window technique. **B** Intraoperative situation after additional application of a resilient resorbable collagen barrier membrane (OSSIX Plus). **C** Palatal image immediately after surgery shows tension-free sutures

**Fig. 3:**
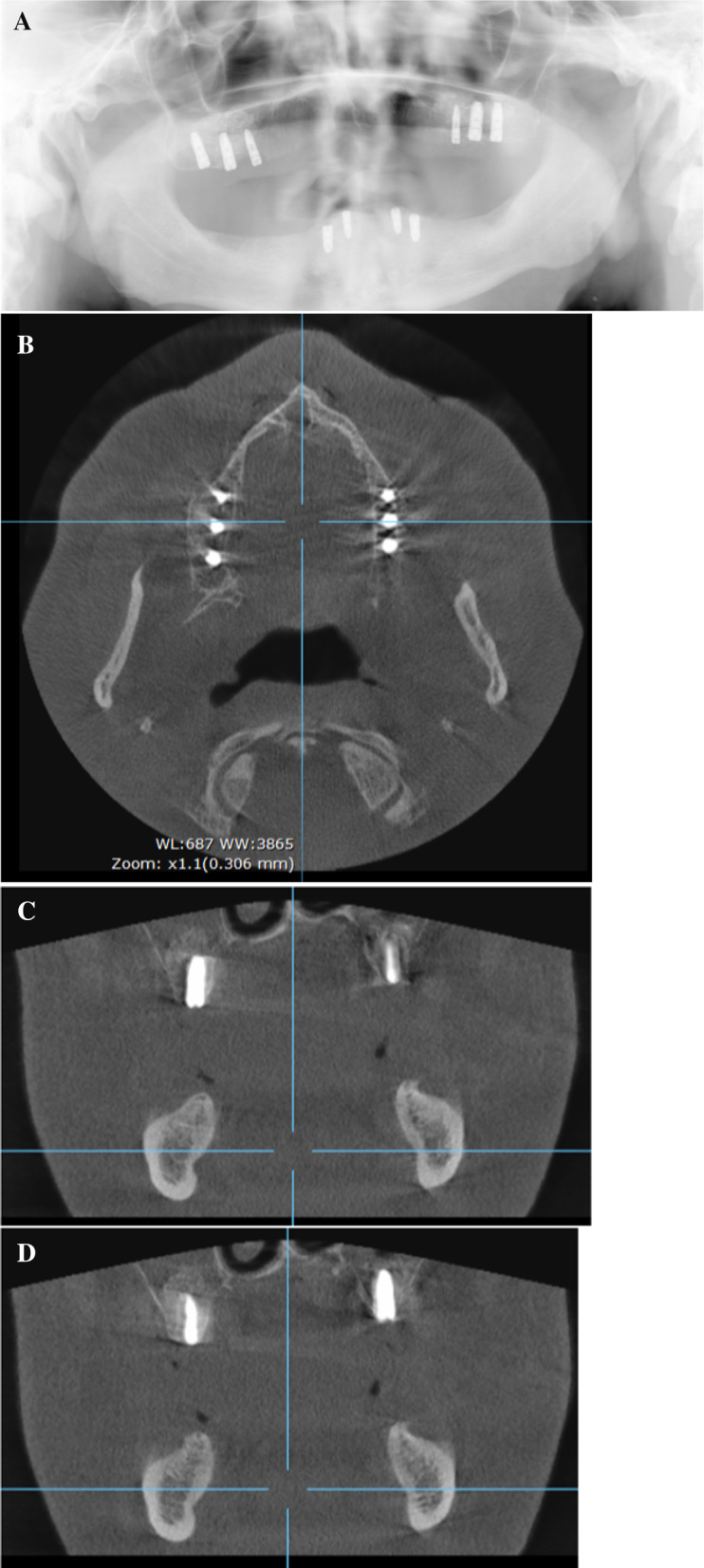
Radiographic evaluation after one-stage lateral sinus lift with simultaneous implantation. **A** Panoramic radiograph directly after alveolar ridge augmentation and simultaneous insertion of six dental implants. **B** Palatinal section of the CBCT illustrating the region of interest. **C** Sagittal section of the CBCT illustrating the region of interest in the first quadrant. **D** Sagittal section of the CBCT illustrating the region of interest in the second quadrant

**Fig. 4:**
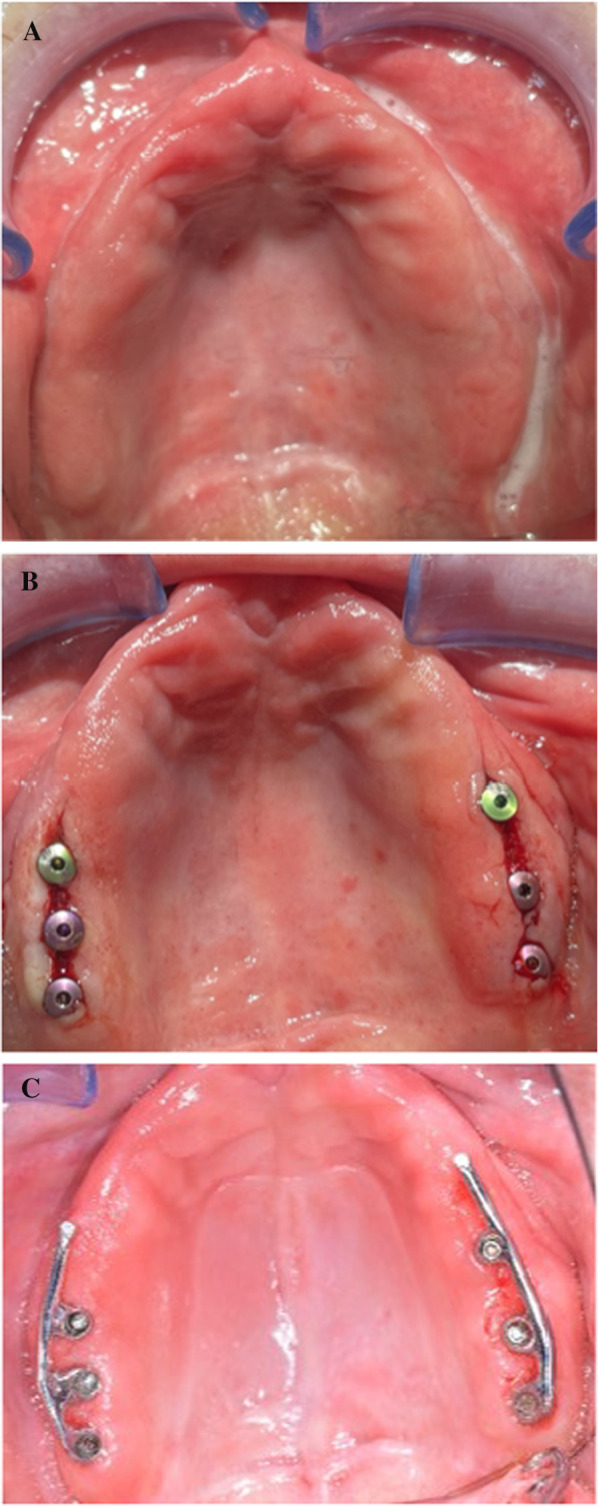
Intraoral image 8 months after surgery. The patient was treated with a removable bar-supported prosthesis. **A** Before exposing the implants. **B** After exposing the implants. **C** Finished bar denture

**Fig. 5:**
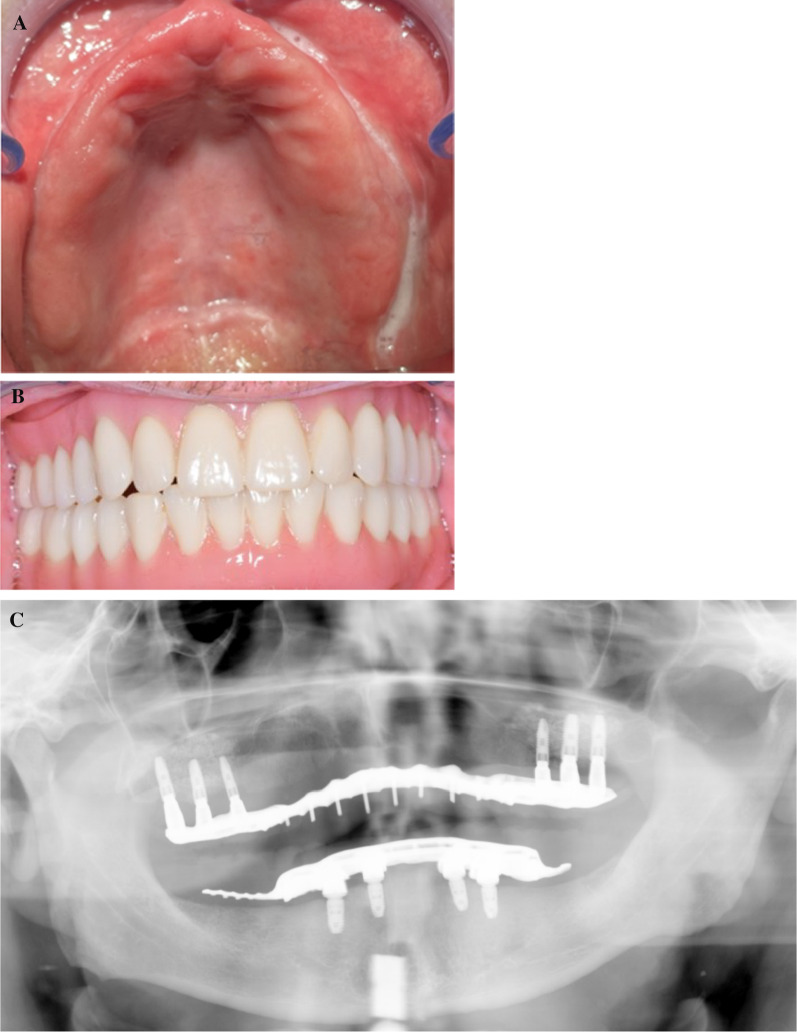
Final overview of restorative dental care. **A** Palatal view of the bar prosthesis in the maxilla. **B** Frontal view of the final situation. **C** Panoramic radiograph showing good integration of the augmentation material and the implants

One case was fully documented to illustrate the surgical procedure and prosthetic restoration. It involved an edentulous man in his late 50s who was to be restored with a bar construction on implants in the maxilla to accommodate a prosthesis. Figure 1 shows the initial radiological situation with a severely atrophied alveolar process of the maxilla. Three implants were to be placed in both the first and second quadrants. Figure 2 demonstrates the intraoperative situation with a lateral approach for sinus lift. The use of a resorbable collagen membrane is also illustrated. A tension-free wound closure could be achieved. Figure 3 shows the radiological findings after augmentation and simultaneous implantation of a total of six dental implants. Figure 4 illustrates the complication-free healing process 8 months after surgery. The implants could be restored very well with a bar construction. Figure 5 shows the final results of the prosthetic restoration. The panoramic X-ray shows complete and stable healing of the implants.

### Demographics of the study population

This study included 63 patients (40 females, 23 males) with an average age of 58.1 ± 11.8 years (minimum: 31 years; maximum: 89 years). The majority of patients were non-smokers (*n* = 57; 90.5%). Only two patients (3.2%) suffered from Diabetes mellitus, and only three patients (4.8%) underwent anticoagulant therapy. One patient received six implants, one patient received four implants, and one patient received three implants. Nearly half of the patients (*n* = 30; 47.6%) was treated with two implants, and the other half of the patients (*n* = 30; 47.6%) received one implant.

The mean follow-up time for implant survival was 80.3 ± 25.9 months (minimum: 38 months; maximum: 145 months; corresponding to an average of 6.7 ± 2.2 years). There was no difference in the follow-up time between the two study groups (Table 1; *p* = 0.232).

### Height of the residual alveolar bone

The height of the residual alveolar bone before sinus lift was on average 2.198 ± 0.577 mm in the severely atrophic study group (< 3 mm) and 3.890 ± 0.966 mm in the reference group (≥ 3 mm). The difference in height between the two study groups was statistically highly significant (*p* < 0.001; Table 1). The minimum height of the residual alveolar ridge was 0.9 mm and the maximum height of the residual alveolar ridge was 7.7 mm.

The bone loss at follow-up was on average 0.310 ± 0.931 mm in the severely atrophic study group (< 3 mm) and 0.288 ± 0.914 mm in the reference group (≥ 3 mm). The difference in bone loss between the two study groups was not significant (*p* = 0.906).

### Implant success rate

Three out of 103 implants were lost during follow-up. Therefore, the overall implant survival rate was 97.09%. One implant was lost in the severely atrophic maxilla group and two implants were lost in the reference group. There was no difference in the occurrence of implant loss between the two study groups (Table 2; *p* = 0.558).

**Table 2:** Complications in dependence of the residual height of the alveolar ridge

	Residual height, binary		*p*-value
	< 3 mm [*n* = 63]	≥ 3 mm [*n* = 40]	
Is the implant still in situ?			
Yes [*n* = 100]	62	38	0.558
No [*n* = 3]	1	2	
Bleeding-on-probing			
No [*n* = 35]	17	18	0.087
Yes [*n* = 68]	46	22	
Probing depth (mm)			
0 mm [*n* = 68]	41	27	0.645
4 mm [*n* = 9]	5	4	
5 mm [*n* = 12]	7	5	
6 mm [*n* = 12]	9	3	
8 mm [*n* = 1]	1	0	
12 mm [*n* = 1]	0	1	
Peri-implantitis			
No [*n* = 88]	54	34	0.999
Yes [*n* = 15]	9	6	
Mucositis			
No [*n* = 19]	11	8	0.797
Yes [*n* = 84]	52	32	
Membrane perforation			
No [*n* = 67]	47	20	0.019
Yes [*n* = 36]	16	20	

*P*-values were obtained from Chi-squared tests

**Fig. 6:**
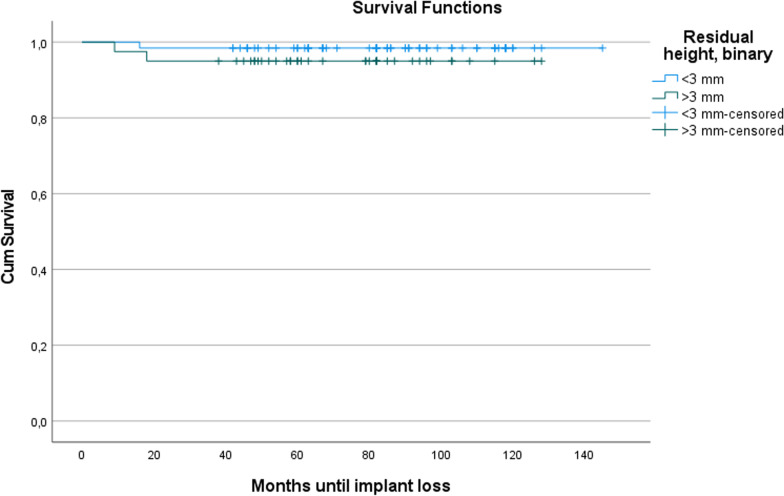
Kaplan–Meier survival curve for the probability of implant loss depending on the residual height of the alveolar ridge (< 3 mm or ≥ 3 mm). The time in months after augmentation and simultaneous implant placement was plotted on the abscissa. The ordinate indicates the cumulative survival probability, i.e., the probability that no implant loss occurred

The Kaplan–Meier estimate for the period in which no implant loss occurred was 142.9 months (95% confidence interval: 138.9–146.9 months) for implants in maxillae with a residual height < 3 mm and 122.3 months (95% confidence interval: 114.5–130.2 months) for implants in maxillae with a residual height greater than 3 mm. Figure 6 shows the corresponding Kaplan–Meier survival curves. The two survival curves were not statistically different from each other (Mantel–Cox test: *p* = 0.317).

### Bleeding-on-probing

Bleeding-on-probing was detected at 68 implants (66.0%). All implants, which exhibited signs of peri-implantitis (*n* = 15), were also positive for the bleeding-on-probing test. However, 53 out of 88 implants (60.2%), which were not affected by peri-implantitis, also showed bleeding-on-probing. Bleeding-on-probing was also associated with the presence of a mucositis (Chi-squared test: *p* = 0.002). Out of 84 implants with a mucositis, 50 implants showed bleeding-on-probing.

There was no difference in the occurrence of bleeding-on-probing between the two study groups (Table 2; *p* = 0.087). In addition, there was no difference in the probing depth between the two study groups (Table 2; *p* = 0.645).

### Peri-implantitis

Fifteen out of 103 implants (14.6%) showed signs and symptoms of a peri-implantitis. There was no association between the occurrence of peri-implantitis and the residual height of the alveolar ridge (Table 2; *p* = 0.999).

### Peri-implant mucositis

An inflammatory lesion of the peri-implant mucosa was detected around 84 out of 103 implants (81.6%). The incidence of peri-implant mucositis was not associated with the residual height of the alveolar ridge (Table 2; *p* = 0.797).

### Membrane perforation

Only 16 out of 63 implantation sites from the severely atrophic study group exhibited membrane perforation (25.4%), while 20 out of 40 implantation sites from the reference group exhibited membrane perforation (50.0%). The difference in the frequency of this complication was statistically significant (Table 2; *p* = 0.019).

## Discussion

This retrospective comparative study aimed to answer the research question whether in very severely atrophied maxillary bones (residual height < 3 mm), a sinus lift with simultaneous implant placement would be associated with a higher complication rate compared to single-stage sinus lifts at average residual alveolar process heights.

Based on the results from this study, it can be concluded that a one-stage lateral sinus lift with simultaneous implantation in severely atrophic maxilla with less than 3 mm of residual bone height was not at risk of a higher complication rate compared to the same procedure in maxillae with residual bone heights of more than 3 mm.

Our findings are in line with similar studies [17, 18]. In a study of 60 partly edentulous patients, who needed 1 to 3 implants and had 1 to 3 mm residual bone height and at least 5 mm bone thickness below the maxillary sinus, no statistically significant differences between implants placed after 1- and 2-step sinus lifting operations were found [17]. Another study compared implantation results following maxillary sinus floor augmentation (MSFA) in 71 edentulous individuals with a remaining alveolar bone height of less than 3 mm applying four different techniques: one-step BAOSFE (bone-added osteotome sinus floor elevation procedure) with immediate implant placement; two-step BAOSFE with subsequent implantation; one-step lateral window sinus lift with immediate implant placement; and two-step lateral window sinus lift with postponed implantation [18]. Despite the limited sample count in each study group, it was concluded that patients with highly atrophic posterior upper jaws might choose one-step and two-step MSFA operations as alternate therapeutic alternatives, based on the positive implantation results [18].

Interestingly, a systematic review on the effect of residual bone height and vertical graft size on new bone formation and graft shrinkage showed that new bone formation was essentially independent of preoperative bone height [19]. On the contrary, the smaller the volume was of the graft placed, the higher the amount of new bone formed, and the smaller the graft shrinkage was [19].

The most frequent intraoperative complication during sinus surgery is damage to Schneider’s membrane [20]. Postoperative complications include wound infection, abscess or dehiscence with drainage, maxillary sinusitis of the surgical site, exposure of the graft and loss of the graft [20, 21]. None of these complications occurred during the study. This finding is in line with a recent meta-analysis that Schneiderian membrane perforation during maxillary sinus floor augmentation procedures with lateral approach is not a risk factor for dental implant survival [22]. However, membrane perforations in maxillary sinus floor augmentation may be significantly reduced applying piezoelectrical devices for MSA [23].

All biomaterials used in this study are well-documented in the current literature and have multiple applications in oral surgery [24], but the application of a one-stage lateral sinus lifting approach with simultaneous implantation for cases with a residual crestal bone height of less than 3 mm has been less investigated. A recent meta-analysis provided moderate evidence that the repositioned bone lid favored the formation of new bone to a higher extent as compared to resorbable membranes [25]. Another solution for limited alveolar bone heights is the usage of short implants. A recent meta-analysis found that there was no evidence that the survival rate of short implants combined with transcrestal sinus floor elevation was lower or higher than that of conventional implants [26].

The implant survival rate was 97.09%. This value lies within the average 10-year survival rate for dental implants as assessed in a recent meta-analysis (96.4%; 95%CI 95.2–97.5%) [27]. Therefore, the implant survival rate of our study was not inferior to the value obtained from the current literature.

Bleeding-on-probing was observed in 66.0% of cases. This value was quite high when compared to the literature, where a bleeding-on-probing probability for a peri-implant site with a probing depth of 4 mm was calculated at 27% [28]. However, other studies also reported higher rates of peri-implant bleeding-on-probing (37.9% in [29]; 39% in [30]). Therefore, this factor needs further investigation. We found that the augmentation material was strongly associated with the presence of bleeding-on-probing. Those implants, where a combination of xenogenic and autogenous was applied for sinus lift, showed a reduced rate of bleeding-on-probing. Interestingly, a comparison of the outcomes of implants inserted in maxillary sinuses augmented with 100% anorganic bovine bone grafts (ABB) versus mixed with 50% ABB and 50% autologous bone graft using a lateral window approach found similar clinical outcomes of implants inserted in sinuses grafted with ABB versus implants inserted in sinuses grafted with mixed 50% ABB and 50% autologous bone [31]. In addition, Pesce and colleagues found that minimizing the augmentation volume might be beneficial to graft healing and stability especially when using allografts and xenografts [19].

Signs and symptoms of a peri-implantitis were detected in 14.6% of implants. The rate of peri-implantitis in our study is higher than that of a recent prospective study that examined implants placed in two-stage maxillary sinus augmentation, which reported a 6.6% prevalence [32]. However, a recent meta-analysis evaluated a total of 2734 subjects and 7849 implants found the prevalence of peri-implantitis to be 17% [33].

A recent multicenter cross-sectional study by Stacchi et al. [34] analyzed factors influencing the prevalence of peri-implantitis in implants inserted in augmented maxillary sinuses. The prevalence of patients presenting peri-implant pathologies was 19.9% [34]. Using different case definitions, Koldsland and coworkers observed significant variations in the prevalence of peri-implantitis within the same patient group (11.3–47.1%) [35].

To the best of our knowledge, our study is the first to use a one-stage lateral sinus lift procedure in severely atrophic maxillae with residual crestal bone height of less than 3 mm. Therefore, the results of our study add a considerable amount of knowledge on the limits of augmentative surgery for oral rehabilitation. However, the monocentric character and the absence of a radiographic evaluation of bone substitute resorption can be considered as limitations of our study. Nonetheless, the large sample number, the ethical approval to our study design and the long follow-up period compensate for the weaknesses and render our study as a valuable contribution to the ongoing discussion on the applicability of one-stage augmentation and dental implantation procedures.

## Conclusion

One-stage lateral sinus lift using autogenic bone chips and xenogenic bone substitutes as filling material and a resorbable collagen membrane as a barrier membrane can be performed as a predictable and effective technique in the treatment of posterior edentulous maxillae with less than 3 mm residual vertical bone height.

## Data Availability

The datasets used and/or analyzed during the current study are available from the corresponding author on reasonable request.
